# IL-17 Produced during *Trypanosoma cruzi* Infection Plays a Central Role in Regulating Parasite-Induced Myocarditis

**DOI:** 10.1371/journal.pntd.0000604

**Published:** 2010-02-16

**Authors:** Paulo Marcos da Matta Guedes, Fredy R. S. Gutierrez, Flavia L. Maia, Cristiane M. Milanezi, Grace K. Silva, Wander R. Pavanelli, João S. Silva

**Affiliations:** Department of Biochemistry and immunology, School of Medicine at Ribeirão Preto, University of São Paulo, Ribeirão Preto, São Paulo, Brazil; René Rachou Research Center, Brazil

## Abstract

**Background:**

Chagas disease is a neglected disease caused by the intracellular parasite *Trypanosoma cruzi*. Around 30% of the infected patients develop chronic cardiomyopathy or megasyndromes, which are high-cost morbid conditions. Immune response against myocardial self-antigens and exacerbated Th1 cytokine production has been associated with the pathogenesis of the disease. As IL-17 is involved in the pathogenesis of several autoimmune, inflammatory and infectious diseases, we investigated its role during the infection with *T. cruzi*.

**Methodology/Principal Findings:**

First, we detected significant amounts of CD4, CD8 and NK cells producing IL-17 after incubating live parasites with spleen cells from normal BALB/c mice. IL-17 is also produced *in vivo* by CD4^+^, CD8^+^ and NK cells from BALB/c mice on the early acute phase of infection. Treatment of infected mice with anti-mouse IL-17 mAb resulted in increased myocarditis, premature mortality, and decreased parasite load in the heart. IL-17 neutralization resulted in increased production of IL-12, IFN-γ and TNF-α and enhanced specific type 1 chemokine and chemokine receptors expression. Moreover, the results showed that IL-17 regulates T-bet, RORγt and STAT-3 expression in the heart, showing that IL-17 controls the differentiation of Th1 cells in infected mice.

**Conclusion/Significance:**

These results show that IL-17 controls the resistance to *T. cruzi* infection in mice regulating the Th1 cells differentiation, cytokine and chemokine production and control parasite-induced myocarditis, regulating the influx of inflammatory cells to the heart tissue. Correlations between the levels of IL-17, the extent of myocardial destruction, and the evolution of cardiac disease could identify a clinical marker of disease progression and may help in the design of alternative therapies for the control of chronic morbidity of chagasic patients.

## Author Summary

Chagas disease is caused by the intracellular parasite *Trypanosoma cruzi*. This infection has been considered one of the most neglected diseases and affects several million people in the Central and South America. Around 30% of the infected patients develop digestive and cardiac forms of the disease. Most patients are diagnosed during the chronic phase, when the treatment is not effective. Here, we showed by the first time that IL-17 is produced during experimental *T. cruzi* infection and that it plays a significant role in host defense, modulating parasite-induced myocarditis. Applying this analysis to humans could be of great value in unraveling the elements involved in the pathogenesis of chagasic cardiopathy and could be used in the development of alternative therapies to reduce morbidity during the chronic phase of the disease, as well as clinical markers of disease progression. The understanding of these aspects of disease may be helpful in reducing the disability-adjusted life years (DALYs) and costs to the public health service in developing countries.

## Introduction

*Trypanosoma cruzi* is an intracellular protozoan parasite that causes Chagas' disease, the major cause of infectious heart disease in Latin America. It is estimated that 13 million people are infected with *T. cruzi* in the Central and South America, and 75 million are at potential risk of infection (WHO, 2005). In non-endemic countries, blood transfusions, organ transplantations, and mother-to-child infection represent real risks for disease transmission, due to high numbers of immigrants and the autochthonous transmission of *T. cruzi* in the USA has been reported [1]. During chronic phase, around 10% and 20% of infected patients develop digestive (megaesophagus and megacolon) and cardiac (cardiomegaly) form of Chagas disease, respectively. The myocarditis that occurs as a result of infection is thought to be due to parasites in the lesions, although immune-mediated mechanisms also appear to be involved in heart pathology [2]. Of note, the immune hyperactivity that is deleterious to the host is governed by the imbalanced production of cytokines in response to the parasite [3].

The pro-inflammatory cytokines IL-12, IFN-γ, and TNF-α act in concert to activate macrophages to kill the parasites through the production of nitric oxide and nitrogen free radicals [4]. In addition, these cytokines also stimulate the differentiation and proliferation of Th1-biased CD4^+^ T cells, which orchestrate a CD8^+^ T-cell response that causes tissue destruction and fibrosis [5]. As expected, the inflammatory response is down-regulated by the anti-inflammatory cytokines IL-10 and TGF-β [6],[7], regulatory T cells [8]–[10], and CTLA-4^+^ cells [11],[12]. Lymphocytes of patients with chronic chagasic cardiopathy (CCC) produce higher amounts of IFN-γ, TNF-α, and IL-6, but little or no IL-4 or IL-10 compared to asymptomatic individuals [3],[13].

For years, the balance of immune inflammation was explained by the dichotomy of cytokines produced. However, the Th1-Th2 paradigm has been reconsidered following the discovery of a novel lineage of effector CD4^+^ T helper lymphocytes, called Th17 cells, which produce interleukin 17 (IL-17)-A and F, IL-21, IL-22, and TNF-α [14]. Th17 differentiation is thought to be mediated by the combined effects of the transcription factors RORγt and RORα, which are dependent on STAT-3, and requires IL-1β, IL-6, IL-21, TGF-β, and the expression of the CCR6 chemokine receptor [15],[16]. In addition to Th17 cells, other cells produce IL-17, including CD8^+^ T cells, γδ T cells, neutrophils, monocytes, and NK cells [17]. IL-17 has pro-inflammatory properties and induces fibroblasts, endothelial cells, macrophages, and epithelial cells to produce several inflammatory mediators, such as GM-CSF, IL-1, IL-6, TNF-α, inducible nitric oxide synthase (iNOS) activation, metalloproteinases, and chemokines (CXCL1, CXCL2, CXCL8, CXCL10), leading to the recruitment of neutrophils and inflammation [18]–[20]. The Th17 response has been linked to the pathogenesis of several inflammatory and autoimmune diseases, such as multiple sclerosis, psoriasis, rheumatoid arthritis, colitis, autoimmune encephalitis [21], schistosomiasis [22], and toxoplasmosis. Infection with *T. cruzi* also leads to the production of several chemokines and cytokines [23] as well as iNOS [4] and metalloproteinase activation [24]. Also, lymphocytes from infected mice and chagasic patients recognize self-epitopes [2], suggesting a possible autoimmune response. Altogether, these observations suggest the possible involvement of IL-17 in the pathogenesis of *T. cruzi* infection. Therefore, we investigated IL-17 production in *T. cruzi*-infected mice and its role in the modulation of the immune response. Our results show that IL-17 is produced during the acute phase of *T. cruzi* infection and controls cardiac inflammation by modulating the Th1 response.

## Methods

### Animals, parasites, and experimental infection

BALB/c female mice, 6 weeks old, were cared for according to institutional ethical guidelines and the Ethics Committee in Animal Research of the FMRP-USP approved all experimental protocols. Mice were infected via the i.p. route with 100 blood trypomastigotes of *T. cruzi*, Y strain. For *in vitro* experiments, the trypomastigotes were grown and purified from a fibroblast cell line (LLC-MK_2_).

### Treatment of mice with mAb against IL-17

Balb/c mice were treated one day before inoculation, and on days four and eight with i.p. injections of 100 µg of normal rat IgG or rat anti-mouse IL-17 (IgG2a, clone M210, Amgen, Seattle, WA). Parasitemia was measured in 5 µL of blood obtained from the tail vein, and mortality was evaluated with five mice per treatment group. Sera, heart tissue, livers, and spleens were collected 14 days p.i from five infected and treated mice.

### Splenocyte cultures

To determine if the parasite induces IL-17 production, naive splenocytes (5×10^6^ cells/ml) from Balb/c mice were cultured for 48 h with trypomastigotes (2.5×10^7^/ml) in 48-well plates (final volume of 0.5 ml) and intracellular IL-17A in CD4^+^, CD8^+^, and NK cells was determined. To shed light on the mechanism that controls IL-17 production, splenocytes (1×10^6^ cells/ml) from normal or *T. cruzi*-infected Balb/c mice (14 days p.i.) were cultured with Con-A (5 µg/ml) (Sigma-Aldrich, St. Louis) and *T. cruzi* antigen (10ηg/well), with or without antibodies against IL-17 or IFN-γ (IgG1, clone R46A2) (10 µg/well), and cytokine production was determined.

### Cytokine quantification (ELISA)

Cytokine production was assayed in sera, tissue heart, liver, and spleen. Tissue fragments were added to vials containing PBS (50 mg/ml) with a protease inhibitor cocktail (Complete, Roche). The tissue fragments were macerated, centrifuged, and the supernatants collected for cytokine quantification. The ELISA sets were IL-1β, IL-4, IL-6, IL-10, IL-12, IL-17, IL-25, IFN-γ, TNF-α, and TGF-β (R&D, Minneapolis, MN), and procedures were performed according to the manufacturers' instructions. Optical densities were measured at 450 ηm. Results are expressed as picograms per milliliter. The limits of sensitivity for the different assays were as follows: IL-4, IL-17, IL-10, TGF-β and TNF-α: 15 pg/ml; IL-12: 10 pg/ml; IFN-γ: 50 pg/ml, IL-1β: IL-25 and IL-6: 20 pg/ml.

### Isolation of leukocytes from cardiac tissue

To isolate mononuclear cells from myocardial tissues, the hearts from five mice were removed at 14 days post infection (p.i.), washed (to remove blood clots), pooled, minced with scissors into small fragments, extensively washed, and subjected to enzymatic digestion with a 500 mg/ml liberase solution (Roche Applied Science, Indianapolis, IN) for 30 min at 37°C. The tissues were processed with RPMI 10% FCS and 0,05% DNAse (Sigma-Aldrich) using Medmachine (BD) for 4 min, the cell suspension was spun, the supernatant removed, and the pellet suspended in RPMI 10% FCS. Suspensions of total spleen cells were washed and the leukocyte purification from these samples and from cardiac cells was done in Ficoll Hypaque (d = 1077 g/ml, Sigma - Aldrich) gradient by centrifuging at 400×g by 30 min at room temperature. The leukocytes obtained were evaluated by flow cytometry.

### Intracellular cytokine detection

The expression of IL-17 and IFN-γ in leukocytes (1×10^6^/well) from heart and spleen were assayed after incubation with monensin (2 µg/ml) for 6 h in RPMI 1640 supplemented with fetal bovine serum (10%). The cells were washed in cold PBS and samples of 5×10^5^ cells/tube were incubated for 30 min at 4°C with 0.5 µg of anti-CD16/CD32 mAb (FC block), followed by the addition of 0.5 µg of PERCP- or FITC-labeled antibodies against CD3, CD4, CD8, or PanNK (all from BD Pharmingen, San Diego, CA) for an additional 30 minutes at 4°C in the dark. To detect intracellular IL-17 and IFN-γ, the cells were fixed with cytofix and cytoperm solution for 15 min at room temperature, washed, and then stained with FITC- or PE-labeled antibodies at 4°C in the dark and incubated overnight. Subsequently, the cells were washed twice and suspended in 200 µL of PBS/1% formaldehyde. For each assay 50,000 events were acquired and data acquisition was performed using a FACSorter. Multivariate data analysis was performed using FlowJo software. The data was exported from the histogram and were processed in Prism Software for statistical analysis and graphics.

### Cardiac parasitism

DNA from the hearts of mice at 14 days p.i. was purified using the SV Total DNA Isolation System kit (Promega, Madison, WI) according to the manufacturer's instructions. Real-time PCR was performed using the Platinum SYBR Green qPCR SuperMix UDG with ROX reagent (Invitrogen, Carlsbad, CA) with 100 ηg of total gDNA. The sequences of primers used were TCZ-F 5′-GCT CTT GCC CAC AMG GGT GC-3′ and TCZ-R 5′-CCAAGCAGCGGATAGTTCAGG-3′. The samples were amplified in a thermal cycler ABI PRISM 7000 Sequence Detection System (Applied Biosystems, Foster City, CA) with the following PCR conditions: first step (2 min at 50°C), second step (10 min at 95°C) and 40 cycles (30 s at 95°C, 30 s at 60°C, and 1 min at 72°C), followed by a dissociation stage. The results were based on a standard curve constructed with DNA from culture samples of *T. cruzi* trypomastigotes (*n* = 3).

### T-bet, GATA-3, Foxp3, STAT-3, RORγt, and chemokine mRNA expression

Total RNA from cardiac tissue was isolated using the TRIZOL reagent (Invitrogen) and SV Total RNA Isolation System (Promega, Madison, WI) according to the manufacturers' instructions. cDNA was synthesized using 1 µg of tRNA through a reverse transcription reaction (ImProm-II^TM^ Reverse Transcriptase, Promega). Real-time PCR quantitative mRNA analyses were performed in an ABI Prism 7000 SDS (Applied Biosystems) using the Platinum SYBR Green qPCR SuperMix UDG with ROX reagent (Invitrogen) for quantification of amplicons. The standard PCR conditions were as follows: 50°C (2 min), 95°C (10 min); 40 cycles of 94°C (30 s), 58°C (30 s), and 72°C (1 min); followed by a standard denaturation curve. The sequences of primers were designed using the Primer Express software package (Applied Biosystems) utilizing nucleotide sequences present in the GenBank database (Table 1). Platinum SYBR Green qPCR SuperMix UDG with ROX reagent (Invitrogen), 1 µg/µl of each specific primer, and a 1:20 dilution of cDNA were used in each reaction. The mean Ct values from triplicate measurements were used to calculate expression of the target gene, with normalization to an internal control (β-actin) using the 2^–*Δ*Ct^ formula.

**Table 1:** Primer sequences and reaction properties.

Target	Sense and Antisense Sequences	bp
B-actin	AGC TGC GTT TTA CAC CCT TT	81
	AAG CCA TGC CAA TGT TGT CT	
Foxp3	ACA ACC TGA GCC TGC ACA AGT	155
	GCC CAC CTT TTC TTG GTT TTG	
T-Bet	CCC ACA AGC CAT TAC AGG ATG	125
	TAT AAG CGG TTC CCT GGC ATG	
GATA-3	AGG AGT CTC CAA GTG TGC GAA	165
	TTG GAA TGC AGA CAC CAC CT	
STAT-3	GCC ACG TTG GTG TTT CAT AAT	58
	GGA ATC GGC TAT ATT GCT GGT	
RORγt	TGG AAG ATG TGG ACT TCG TTT	55
	TGG TTC CCC AAG TTC AGG AT	
CCR3	GCT CTC TGG ATT GAA GTG TGC A	82
	AAG TAT CAC GTC CAC CAC CTG G	
CCR4	CGA TTC CAA AGA TGA ATG CCA	127
	TCC CCA AAT GCC TTG ATA CC	
CCR5	TGC ACA AAG AGA CTT GAG GCA	191
	AGT GGT TCT TCC CTG TTG GCA	
CCR6	GCC CAG CAC ATC ATA GCA TT	52
	CCA GGA TTT GTA AGT TGC CC	
CCL2	AGG ACA GAT GTG GTG GGT TT	53
	TGC AGC AGT CAA CAC AAA TTG	
CCL3	TTC TGC TGA CAA GCT CAC CCT	117
	ATG GCG CTG AGA AGA CTT GGT	
CCL4	CCT GAC CAA AAG AGG CAG ACA	169
	AGC AAG GAC GCT TCT CAG TGA	
CCL5	TTC CCT GTC ATC GCT TGC TCT	101
	CGG ATG GAG ATG CCG ATT TT	
CCL11	AGA TGC ACC CTG AAA GCC AT	107
	TTT GGT CCA GGT GCT TTG TG	
CCL17	GAA GTC CCT GTT CCC TTT TTT	57
	TGT GTT CGC CTG TAG TGC ATA	
CCL20	TTT GGG ATG GAA TTG GAC AC	51
	ACC CCA GCT GTG ATC ATT TC	
CCL22	ATG GTG CCA ATG TGG AAG A	68
	TAA ACG TGA TGG CAG AGG GT	
CXCL9	AAT TTC ATC ACG CCC TTG AGC	90
	CAG CTG TTG TGC ATT GGA TAG C	
CXCL10	AGC GTT TAG CCA AAA AAG GTC	51
	TGG CTT CAC TCC AGT TAA GGA	
*T. cruzi*	GCT CTT GCC CAC AMG GGT GC	195
	CCA AGC AGC GGA TAG TTC AGG	

bp: base pairs of amplicon size.

### Quantification of heart infiltrating cells

The total number of nucleated cells was counted in fifty microscopic fields in at least four representative, nonconsecutive, HE-stained sections (5 µm thickness) from each mouse. Sections were examined using a Zeiss Integrationsplatte II eyepiece (Zeiss Co, Oberkochen, Germany) reticule, using a microscope at a final magnification of 400X.

### Statistical analysis

Data are expressed as means ± SEM. Student's t test was used to analyze the statistical significance of the observed differences in infected *vs*. control assays. In time course studies, one-way ANOVA was used followed by Tukey-Kramer post-hoc analysis. The Kaplan-Meier method was used to compare survival curves of the studied groups. All analyses were performed using PRISM 3.0 software.

## Results

### *T. cruzi* induces IL-17 production

**Figure 1:**
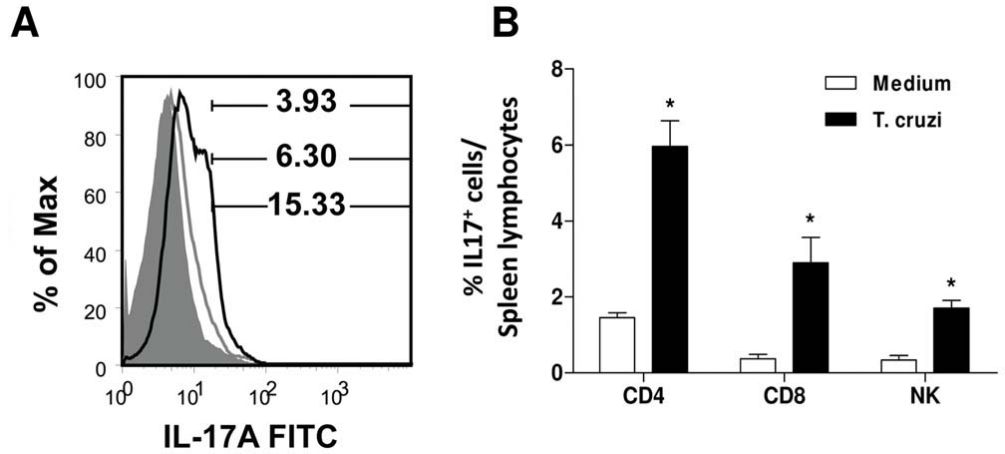
Trypomastigotes of *T. cruzi* induce IL-17 production by mouse splenocytes *in vitro*. Leukocytes from BALB/c mice (5×10^6^ cells/ml) were cultured with or without trypomastigotes (2.5×10^7^/ml) of *T. cruzi* for 48 hours and the intracellular expression of IL-17 determined in total splenocytes (**A**), CD4^+^, CD8^+^, and NK cells (**B**) (mean ± SEM of cultures in triplicate, 3 animals/group). In **A**, the empty histograms represent IL-17^+^ cells harvest from cultures in the presence (black line) or absence of parasites (gray line) and the full histogram represents an IgG FITC control. The data are representative of two independent experiments. * *P*<0.05 compared to uninfected mice.

**Figure 2:**
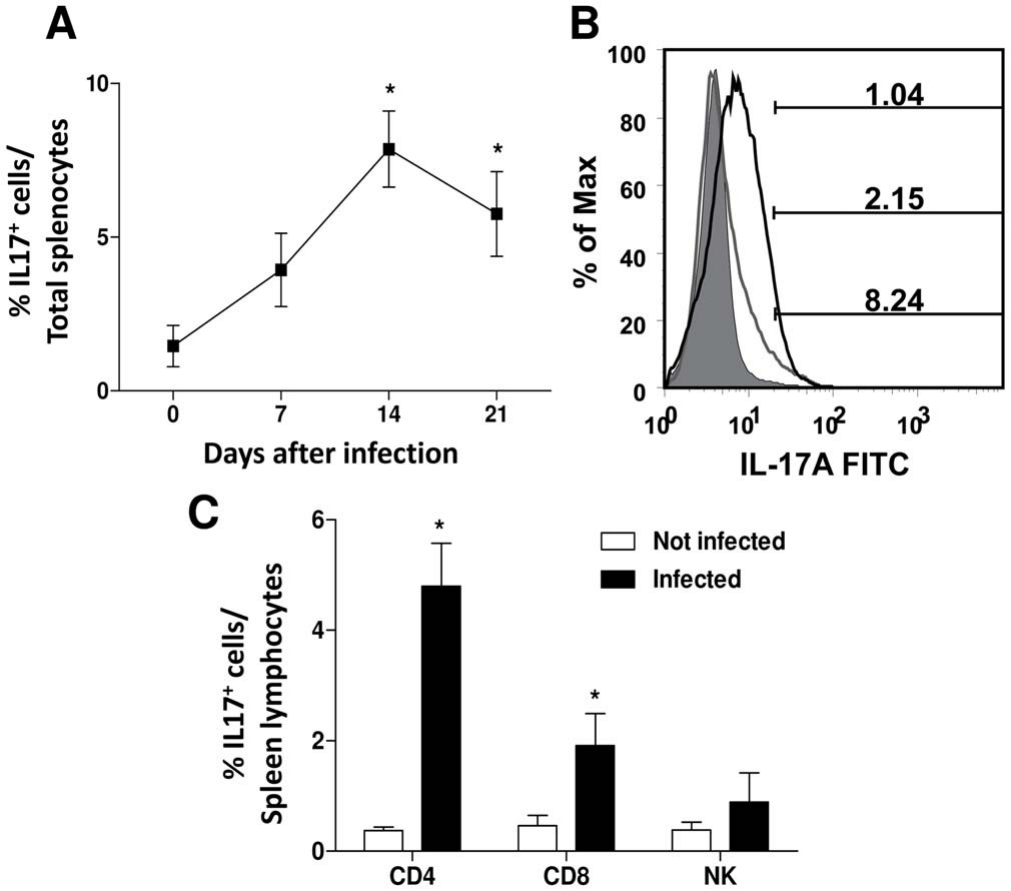
*Trypanosoma cruzi* infection induces IL-17 expression in mouse splenocytes. The *ex vivo* frequencies of IL-17^+^ cells were determined in total splenocytes (**A**) of BALB/c mice on 0, 7, 14, and 21 days p.i.. In **B** and **C**, the percentage (mean ± SEM) of IL-17^+^ lymphocytes and CD4^+^, CD8^+^, NK cells of mice on day 14 p.i. are shown. The data are representative of two independent experiments with 3 mice in each day of infection. * *P*<0.05 compared to uninfected mice.

First, we evaluated IL-17 production by spleen cells from naive mice incubated with live trypomastigotes. We found that the percentage of IL-17^+^ splenocytes in cultures containing *T. cruzi* (13.86±4.59%) was higher than in the controls (5.45±2.87%) (Figure 1A). The mean fluorescence intensities of intracellular IL-17 staining in the absence (6.02±1.15) and presence of parasites (11.29±1.54) indicated that culture with parasites increased the degree of IL-17 production. The major IL-17 expressing cells were CD4^+^ and CD8^+^ T lymphocytes as well as NK cells (Figure 1B). IL-17^+^ cells were significantly increased in spleens of mice during the course of acute infection (Figure 2A). The percentage of IL-17^+^ lymphocytes on day 14 p.i. was also significantly increased compared with uninfected control mice (Figure 2B). On day 14 p.i., the main IL-17^+^ lymphocytes were CD4^+^ (4.79±0.87) followed by CD8^+^ T cells (1.92±0.79) (Figure 2C). These results clearly indicate that infection with *T. cruzi* leads to IL-17 production.

### Blockade of IL-17 increases susceptibility to *T. cruzi* infection

**Figure 3:**
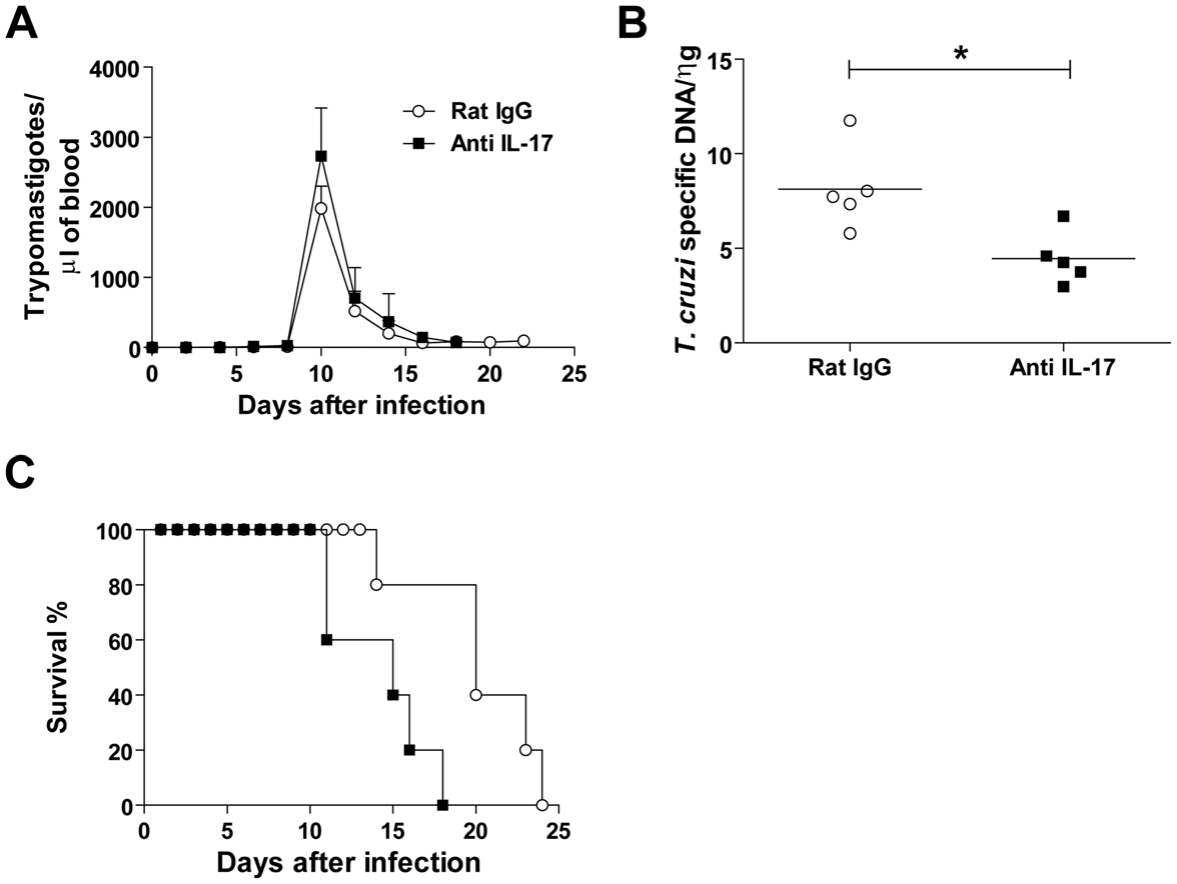
Enhanced cardiac parasitism and mortality in *T. cruzi* infected mice treated with anti-IL-17. Parasitemia (**A**), cardiac parasitism (**B**), and survival (**C**) were determined in mice infected with *T. cruzi.* The quantification of genomic DNA was determined by real time PCR on tissue heart of mice on day 14 p.i. The data (mean ± SEM) are representative of two independent experiments (five mice per group). * *P*<0.05 compared to infected mice treated with normal rat IgG.

**Figure 4:**
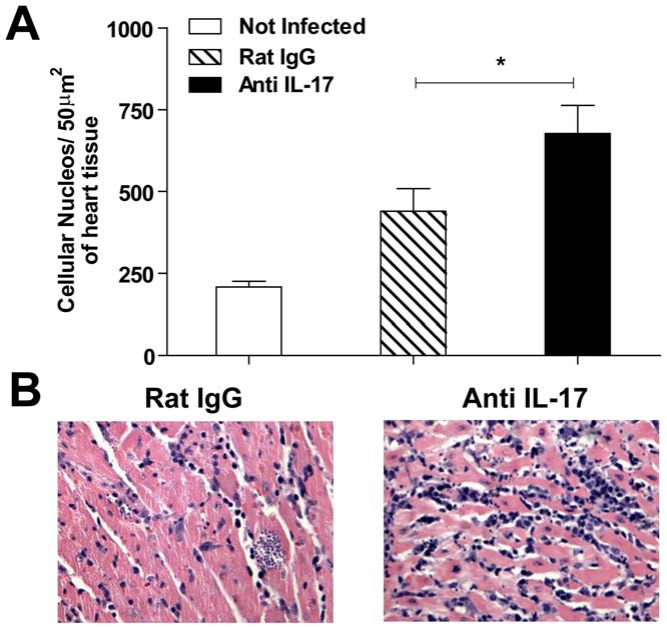
IL-17 neutralization increases *T. cruzi*-induced heart pathology. Quantification of nuclei (**A**) in the heart tissue of normal mice (white bar) and infected mice (14 days p.i.) treated with normal rat IgG (gray bar) or with anti-IL-17 (black bar). In (**B**), representative microphotographs (original magnification ×400) of cardiac tissue of mice on day 14 post *T. cruzi* infection are shown. Data (mean ± SEM) are representative of two independent experiments using five mice per group. * *P*<0.05 compared to infected mice treated with normal rat IgG.

Since IL-17 is produced during the acute phase of *T. cruzi* infection, we investigated its role in parasite control. We treated mice with anti-IL-17 neutralizing antibody and evaluated the course of infection and mortality. We did not find significant differences in the course of parasitemia of anti-IL-17 treated mice compared with the control group (Figure 3A). However, there was a significant reduction in cardiac parasitism of anti-IL-17 treated mice (day 14 p.i.) compared with infected mice treated with normal rat IgG (Figure 3B). Importantly, infected mice treated with anti-IL-17 exhibited significantly earlier mortality compared to controls. The anti-IL-17 treated mice survived only until day 18 p.i., whereas the control group survived until 24 days p.i. (Figure 3C). Inhibition of IL-17 also resulted in more inflammatory cells in the heart tissue of *T. cruzi*-infected mice on day 14 p.i. (Figure 4A and B). The total number of nuclei per 50 µm section of heart tissue was higher in infected mice treated with anti-IL-17 (677.25±89.36) than in controls (439.75±54.87) (Figure 4A). It is noteworthy that by microscopy analysis, the inflammatory infiltrate found in all infected mice was characterized by mononuclear cells, and scarce presence of polymorphonuclear cells, and no difference were detected in the cellular composition of the myocarditis between the groups at day 14 pi. We conclude that IL-17 plays a role in resistance to the infection, modulating the inflammatory reaction that occurs in infected mice.

### Cytokine production in *T. cruzi*-infected mice treated with anti-IL-17

**Figure 5:**
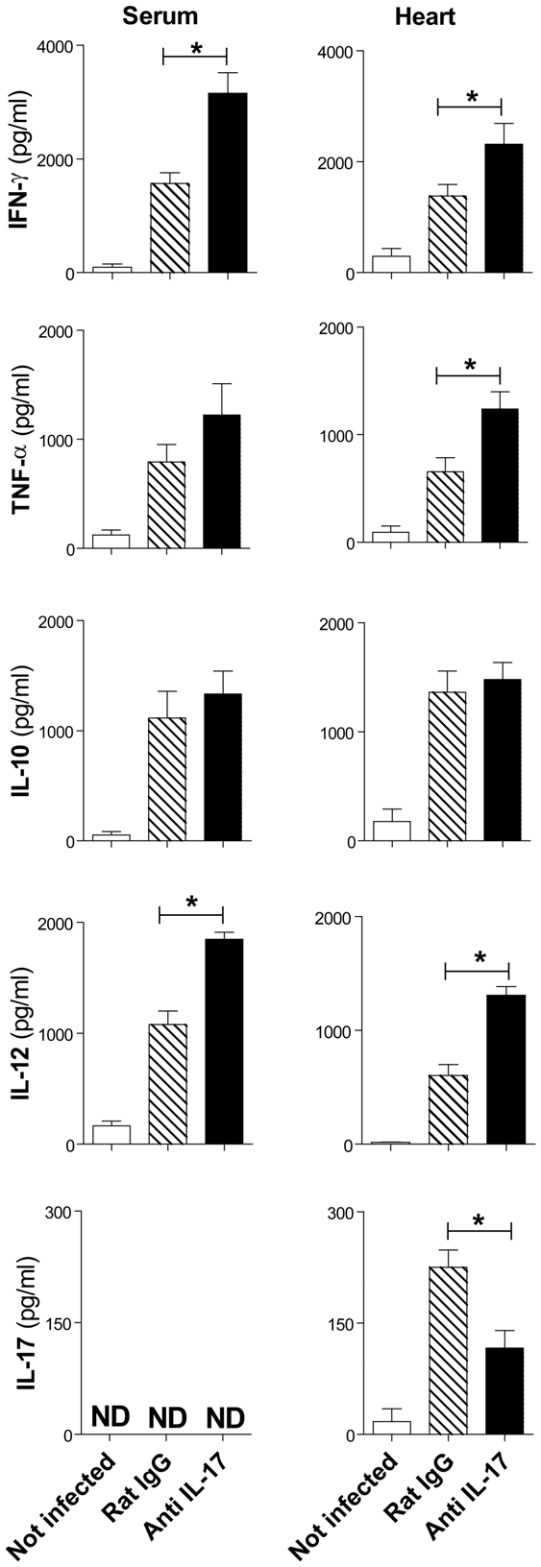
IL-17 controls IFN-γ, TNF-α, IL-12, and IL-17 production in heart tissue of mice infected with *T. cruzi*. IFN-γ, TNF-α, IL-10, IL-12, and IL-17 were quantified in the sera and heart tissue of infected mice (14 days p.i.) that were treated or not with anti-IL-17. For heart cytokine quantification, 50 mg of tissue was homogenized in 1.0 ml of PBS plus protease inhibitors. The data (mean ± SEM) are representative of two independent experiments (five mice per group). * *P*<0.05 compared to infected mice treated with normal rat IgG.

**Figure 6:**
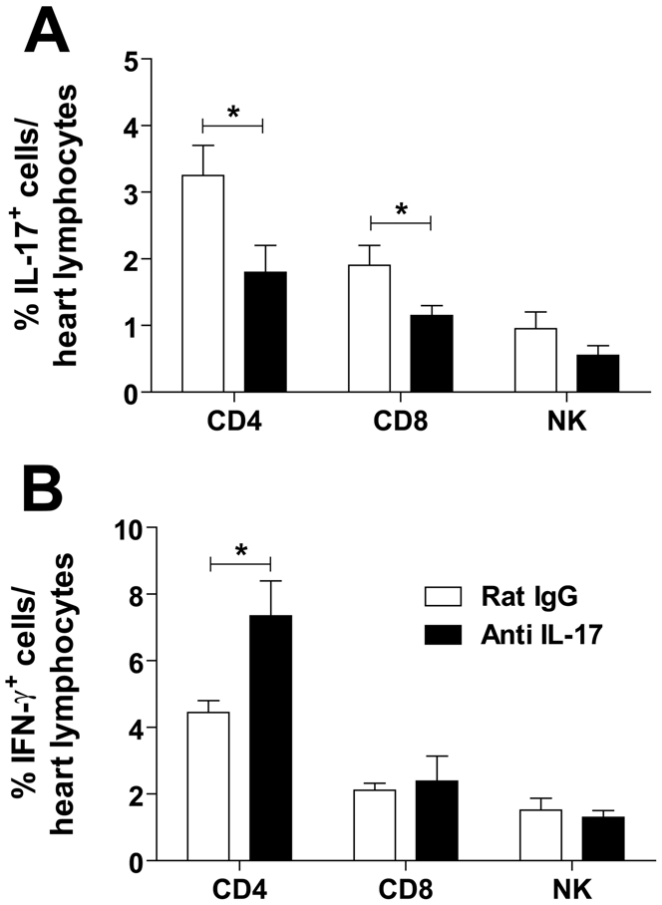
IL-17 controls Th1 differentiation in *T. cruzi*-infected mice. BALB/c mice were infected, the heart harvested on day 14 p.i., the leukocytes isolated and incubated with monensin for 6 h in culture medium, and the intracellular expression of IL-17 (**A**) and IFN-γ (**B**) were determined. Data (mean ± SEM) are representative of two independent experiments using five mice per group. * *P*<0.05 compared to infected mice treated with normal rat IgG.

To investigate the mechanism by which IL-17 neutralization changes the course of infection, we first compared cytokine production in *T. cruzi*-mice treated or not with anti-IL-17. As expected, we detected significant levels of IFN-γ, TNF-α, IL-10, and IL-12 in the sera and tissue heart of *T. cruzi-*infected mice (Figure 5). Inhibition of IL-17 resulted in significantly higher levels of IFN-γ and IL-12 in the sera and heart tissue, and increased TNF-α level in the heart tissue of infected mice compared with mice treated with normal rat IgG. Treatment with anti-IL-17 did not, however, change the levels of IL-10. IL-17 was not detected in the sera but, consistent with previous results, infection increased IL-17 levels in the heart tissue (Figure 5), where it was produced by CD4^+^, CD8^+^ and NK cells (Figure 6A). Again, treatment with anti-IL-17 decreased IL-17 levels (Figure 5) and production by CD4^+^ and CD8^+^ cells in the heart (Figure 6A), and increased IFN-γ production by CD4^+^ cells (Figure 6B). IL-17 neutralization did not changed the frequency of CD4^+^ (29.32±2.14 *vs* 24.37±1.87%), CD8^+^ (52.01±5.38 *vs* 43.52±2.90%) and NK (0.7±0.12 *vs* 1.01±0.19%) cells in the heart. Low levels of IL-25 were detected in the heart tissue and spleens of normal mice, but not in infected mice. IL-1β, IL-4, IL-6, and TGF-β were detected in the sera, myocardium, spleens, and livers of *T. cruzi*-infected mice, but did not change with anti-IL-17 treatment (data not shown).

### IL-17 controls IFN-γ and TNF-α during the acute phase of *T. cruzi* infection

**Figure 7:**
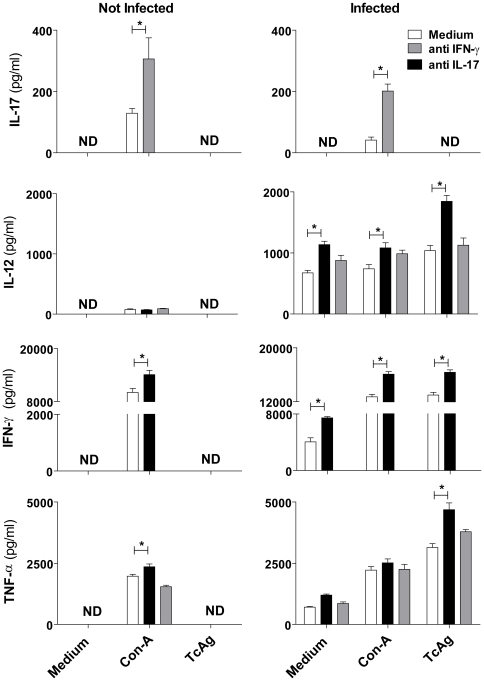
Cross-regulation of IL-12, IFN-γ, and IL-17 during *T. cruzi* infection. Splenocytes from BALB/c mice infected (14 days p.i.) or not with *T. cruzi* were harvested, cultured for 72 hours with or without Con-A (5 µg/ml), *T. cruzi* antigen (10 ηg/well), anti-IL-17 (10 µg/well) or anti-IFN-γ (10 µg/well). The cytokines IL-17, IL-12, IFN-γ, and TNF-α were determined by ELISA. The data show the mean ± SEM of triplicate cultures with 3 mice per group and are representative of two independent experiments. * *P*<0.05 compared to control culture.

Next, we investigated the mechanisms that regulate IL-17 production and the means by which IL-17 modulates Th1 cytokine production. IL-17 was detected only when leukocytes from normal or infected mice were cultured with Con-A, and it was significantly increased when endogenous IFN-γ was inhibited (Figure 7). IL-12, IFN-γ, and TNF-α production by leukocytes from acutely infected mice were also regulated by IL-17, as addition of anti-IL-17 to the cultures significantly increased the production of these cytokines in the presence of parasite antigen. In the presence of mitogen, we found increased production of IL-12 and IFN-γ by the addition of anti-IL-17 (Figure 7). These results show that IL-17 produced during *T. cruzi* infection modulates IFN-γ, TNF-α, and IL-12 production.

### IL-17 neutralization during *T. cruzi* infection increases Th1 responses

**Figure 8:**
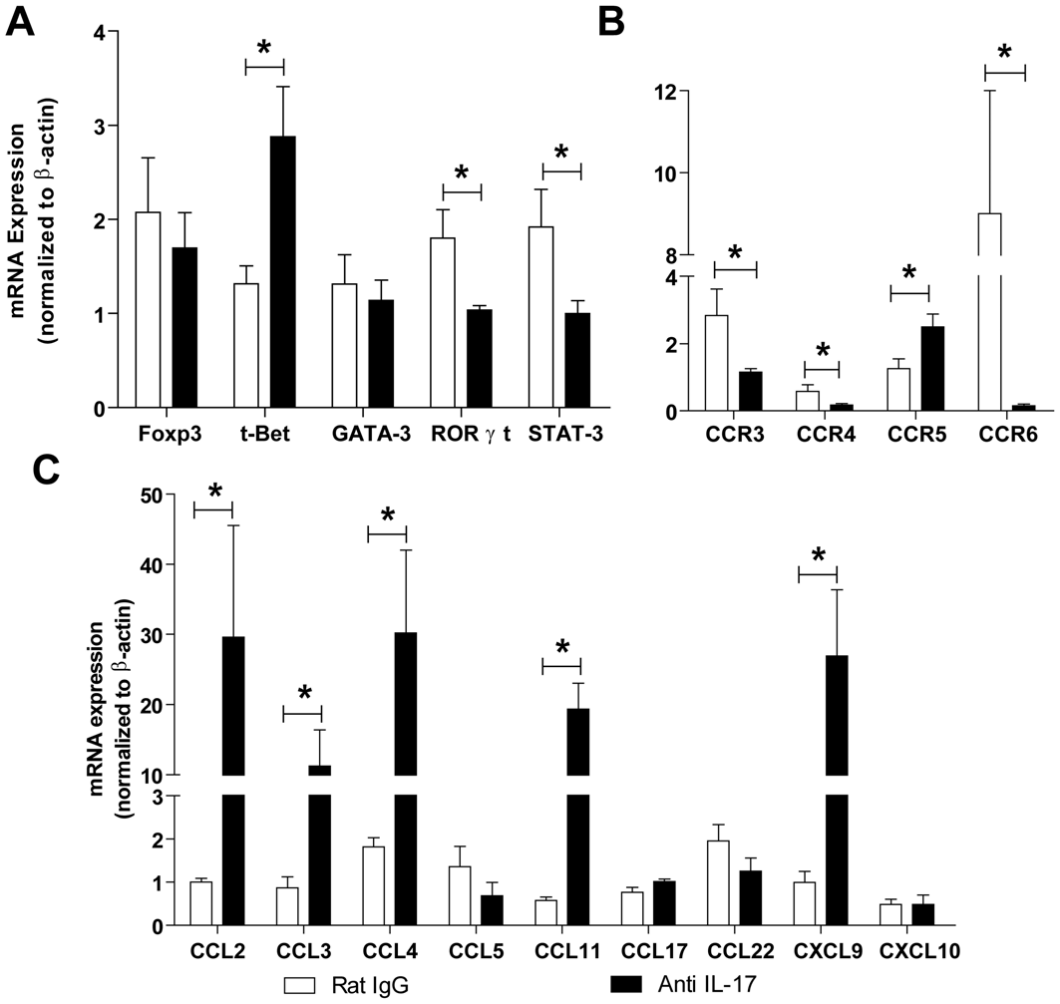
IL-17 controls Th1 differentiation and the expression of chemokines and their receptors in the hearts of *T. cruzi*-infected mice. mRNA expression of transcription factors (**A**), chemokine receptors (**B**), and chemokines (**C**) was determined in the heart tissue of *T. cruzi* infected mice (14 days p.i) treated with normal rat IgG or anti-mouse IL-17. The data (mean ± SEM) are representative of two independent experiments (five mice per group). * *P*<0.05 compared to infected mice treated with normal rat IgG.

To understand how IL-17 regulates the pattern of cytokine production, we examined the expression of the transcription factors Foxp3, T-bet, GATA-3, and STAT-3/RORγt. *T. cruzi* infected mice showed enhanced of Foxp3, T-bet, GATA-3, STAT-3, and RORγt mRNA expression in heart tissue compared to uninfected animals. IL-17 neutralization in *T. cruzi*-infected mice significantly increased T-bet but decreased STAT-3 and RORγt mRNA expression in the heart when compared to infected control animals (Figure 8A). Concomitant to the enhanced expression of the Th1 transcription factor (T-bet) and reduction of Th17 transcription factors (STAT-3 and RORγt), the cardiac tissue of IL-17 neutralized mice exhibited higher expression of CCR5, and decreased expression of CCR3 and CCR4 chemokine receptors, both involved in Th2 and regulatory T cell migration [25],[26]. The expression of CCR6, a chemokine receptor expressed by Th17 cells, was markedly reduced in the heart tissue of *T. cruzi* infected mice treated with anti-IL-17 (Figure 8B). The expression of CCL2, CCL3, CCL4, CCL11, and CXCL9, which are involved in T-cell migration to the heart tissue of infected mice, but not CCL5, CCL17, CCL22, or CXCL10, was increased in the heart tissue of anti-IL-17 treated mice (Figure 8C). Therefore, IL-17 inhibits T-bet and Th1 chemokine expression in the heart tissue of *T. cruzi* infected mice.

## Discussion

The results shown here demonstrate that *T. cruzi* infection results in the production of IL-17, which regulates the immune response as well as the development of heart lesions during the course of infection. Moreover, IL-17 regulates the expression of the transcription factors T-bet, RORγt, and STAT-3, the production of inflammatory cytokines and chemokines, and the expression of their receptors in the heart. First, we showed that trypomastigotes induce IL-17 production by CD4^+^, CD8^+^, and NK cells from naïve mice, and that an increased number of IL-17^+^ cells is observed during the acute phase of infection. Low IL-17 production was previously described for splenic CD4^+^ T cells from infected mice cultured with *T. cruzi* antigen [27]. In vivo the amount of leukocytes expressing IL-17 is very relevant, mainly if we consider that the number of lymphocytes increase dramatically during the infection [28]. The mechanism by which the parasites can trigger IL-17 production is unknown, but it could involve antigen recognition by pathogen associated pattern receptors. Parasite compounds lead to the production of IL-12 [29], TNF-α [30], and IL-10 [31], and they activate TLR2 [32], TLR4 [33], and TLR9 [34]. The glycoinositolphospholipid from *T. cruzi* is a TLR4 agonist with proinflammatory effects [35], and TLR4 activation is important for Th17 cell survival through the induction of IL-23 production by dendritic cells [36]. Moreover, mice deficient in TLR4 have markedly lower numbers of Th17 cells and a reduced capacity to produce IL-17 in an experimental model of arthritis [37]. IL-17 production and Th17 differentiation can be induced by IL-1β, IL-6, and TGF-β[15], which are all produced during *T. cruzi* infection [7],[38],[39]. Additionally, the phagocytosis of apoptotic leukocytes [40], a frequent occurrence in *T. cruzi*-infected mice [41], induces TGF-β synthesis [7],[42] and IL-6 production [39], thereby setting the stage for Th17 differentiation. Thus, it is possible that IL-17 production and Th17 driving do occur early after innate immune recognition of *T. cruzi*. We are currently performing additional experiments to study the possible role of TLR2 and TLR4 in *T. cruzi*-driven IL-17 production showed herein. Since trypomastigotes induced IL-17 production by spleen cells in vitro, it is possible that other strains with different tropism also induce similar amount of this cytokine production. However, this important question has to be addressed.

Neutralization of IL-17 using an anti-IL-17 monoclonal antibody [43] did not change the parasitemia of *T. cruzi*-infected mice but resulted in decreased parasitism in heart tissue, and led to earlier mortality. As the Y strain is infective to several cell types, many tissues besides heart can act as parasite reservoirs and consequently can contribute to the peripheral blood parasitemia. IL-17 neutralization also resulted in increased production of IFN-γ and TNF-α in tissues and sera; both cytokines are known to activate macrophages to produce nitric oxide and promote the killing of intracellular amastigotes [4]. In fact, neutralization of IL-17 resulted in significantly less parasite DNA in the heart tissue, probably due to an increase in iNOS production (data not shown). Unlike in rheumatoid arthritis, colitis, and autoimmune encephalitis (EAE) [21], where IL-17 induces and sustains inflammation, in *T. cruzi* infection, IL-17 exhibits a regulatory effect. Some Th17 cells generated via TGF-β and IL-6 [15], cytokines produced during *T. cruzi* infection [7],[39], produce high levels of IL-10 and prevent lesions in EAE [44], consistent with our data suggesting an anti-inflammatory role for IL-17. In accordance, scarce neutrophils were found in the inflammatory substrate of heart of *T. cruzi*-infected mice, independent of treatment or not with anti-IL-17. Our data also show that IL-17 controls IFN-γ production, as previously observed in mice with EAE [45].

To further understand the regulatory effects of IL-17, cultures of spleen cells from normal or infected mice were treated with anti-IL-17. As observed *in vivo*, the cells exhibited increased intracellular expression of IL-12, IFN-γ, and TNF-α, confirming a regulatory role for IL-17 in the immune response of *T. cruzi* infected mice. Since a high level of IFN-γ and TNF-α production can generate undesirable side effects [11],[30],[31], IL-17 could be important in modulating these cytokines during acute *T. cruzi* infection, resulting in fewer heart lesions and delayed mortality. The increased IFN-γ production observed in mice treated with anti-IL-17 could be due to enhanced IL-12 secretion and increased T-bet expression, a transcription factor crucial for Th1 differentiation [46]. Therefore, IL-17 seems to act indirectly on the differentiation of Th1 lymphocytes through the control of IL-12 production. In contrast, the expression of RORγt and STAT-3, which lead to Th17 differentiation [15], were decreased in infected mice treated with anti-IL-17. Analogous to Th1 and Th2 cell responses, Th17 cell responses are amplified by a positive feedback loop [47]. The expression of GATA-3, a Th2 transcription factor [48], remained unchanged. Of note, *T. cruzi* infection induces markedly increased expression of RORγt and STAT-3, clearly indicating the induction of Th17 differentiation.

One important question regards the mechanism of IL-17 modulation of the migration of inflammatory cells to the heart tissue of infected mice. Chemokines, including the ligands of CCR5 [49] and CCR2 [50],[51], are important to the mechanism that leads to myocarditis. In the hearts of mice treated with anti-IL-17, we found increased expression of CCL2, CCL3, CCL4, CXCL9, and CCL11, which, except for the last, clearly favor the Th1 type response. As the expression of chemoattractants is under the control of IFN-γ and TNF-α, it is possible that increased IFN-γ and TNF-α production, as a result of IL-17 neutralization and elevated IL-12 production, is responsible for the increased myocarditis. Accordingly, we found decreased expression of CCR3 (a Th2-associated receptor) and increased expression of CCR5, a receptor crucial for the development of myocarditis [49]. This may explain the decrease in heart tissue parasitism, since the chemokines CCL2, CCL3, CCL4, CCL5, and CXCL9 as well as IFN-γ can induce iNOS activation in macrophages and cardiac myocytes [51],[52]. Also, since CCR4 and CCR6, but not CCR5, are expressed by Th17 cells [53], the decreased expression of CCR4 and CCR6 but increased expression of CCR5 clearly indicates a reduction in Th17 cells in the inflammatory lesions, confirming that treatment with anti-IL-17 was effective.

In summary, our results show that IL-17 plays a role in the pathogenesis of *T. cruzi*-induced myocarditis. We propose that IL-17 elicited during the infection reduces IL-12 production and T-bet expression, which, as a consequence, decreases the production of IFN-γ, TNF-α, and chemokines. Therefore, IL-17 controls Th1 differentiation in *T. cruzi*-infected mice. The role of IL-17 in the pathogenesis of Chagas' disease is a question that deserves further investigation. Correlations between the levels of IL-17, the extent of myocardial destruction, and the evolution of cardiac disease could identify a clinical marker of disease progression and may help in the design of alternative therapies for the control of chronic morbidity of chagasic patients.
